# Ultrasound-Guided Infiltrative Treatment Associated with Early Rehabilitation in Adhesive Capsulitis Developed in Post-COVID-19 Syndrome

**DOI:** 10.3390/medicina59071211

**Published:** 2023-06-28

**Authors:** Danilo Donati, Fabio Vita, Roberto Tedeschi, Stefano Galletti, Alessandro Biglia, Tommaso Gistri, Pasquale Arcuri, Flavio Origlio, Francesco Castagnini, Cesare Faldini, Davide Pederiva, Maria Grazia Benedetti

**Affiliations:** 1 Physical Therapy and Rehabilitation Unit, Policlinico di Modena, 41125 Modena, Italy; 2 Clinical and Experimental Medicine PhD Program, University of Modena and Reggio Emilia, 41121 Modena, Italy; 3 Department of Orthopedic and Traumatological Surgery, IRCCS Istituto Ortopedico Rizzoli, University of Bologna, 40136 Bologna, Italy; 4 Department of Biomedical and Neuromotor Sciences, Alma Mater Studiorum, University of Bologna, 40136 Bologna, Italy; 5 Musculoskeletal Ultrasound School, Italian Society for Ultrasound in Medicine and Biology, 40136 Bologna, Italy; 6 Rheumatology Department, Fondazione IRCCS Policlinico San Matteo, University of Pavia, 27100 Pavia, Italy; 7 Family Medicine Department, ASL Tuscany, 55049 Viareggio, Italy; 8 Physical Therapy and Rehabilitation Unit, IRCCS Istituto Ortopedico Rizzoli, University of Bologna, 40136 Bologna, Italymariagrazia.benedetti@ior.it (M.G.B.)

**Keywords:** ultrasound infiltrative treatment, early rehabilitation, adhesive capsulitis, post-COVID-19 syndrome

## Abstract

*Background and Objectives:* Post-COVID-19 syndrome is commonly used to describe signs and symptoms that continue or develop after acute COVID-19 for more than 12 weeks. The study aimed to evaluate a treatment strategy in patients with adhesive capsulitis (phase 1) developed in post-COVID-19 syndrome. *Materials and Methods*: The method used was an interventional pilot study in which 16 vaccinated patients presenting with the clinical and ultrasound features of adhesive capsulitis (phase 1) developed during post-COVID-19 syndrome were treated with infiltrative hydrodistension therapy under ultrasound guidance associated with early rehabilitation treatment. *Results:* Sixteen patients with post-COVID-19 syndrome treated with ultrasound-guided infiltration and early rehabilitation treatment showed an important improvement in active joint ROM after 10 weeks, especially in shoulder elevation and abduction movements. The VAS mean score before the treatment was 6.9 ± 1.66. After 10 weeks of treatment, the VAS score was 1 ± 0.63. *Conclusions:* The study demonstrated that the management of adhesive capsulitis (phase 1) developed in post-COVID-19 syndrome, as conducted by physiotherapists in a primary care setting using hydrodistension and a rehabilitation protocol, represented an effective treatment strategy.

## 1. Introduction

Post-COVID-19 syndrome includes a set of signs and symptoms accumulated during or after COVID-19 infection that persist for more than 12 weeks and are not explained by an alternative diagnosis [1]. Muscle fatigue or weakness is the most common symptom, reported in 17.5–72% of post-COVID-19 cases, followed by residual dyspnea, with an incidence ranging from 10 to 40%, then chest pain, mental problems such as brain fog, joint pain, and loss of muscle mass [1,2]. In a letter to the editor, Vitali et al. observed an increased prevalence of adhesive capsulitis during the COVID-19 pandemic and suggested three mechanisms causing the disease:

- lack of appropriate physical therapy, leading to painful rigidity of the shoulder;
- depression and/or anxiety due to the COVID-19 pandemic;
- mechanisms relating to extrapulmonary manifestations of COVID-19 [3].

The study of Castro et al. showed an increase in the proportion of magnetic resonance imaging findings suggestive of adhesive capsulitis during the COVID-19 pandemic [4]. In the study by Ascani et al., it was shown that adhesive capsulitis in post-COVID-19 syndrome may be due to the direct and indirect effects of the SARS-CoV-2 virus [1]. Ascani et al. reported 12 cases of adhesive capsulitis in patients after COVID-19 infection where the direct effects of the SARS-CoV-2 were on the synovium and fibroblasts and, therefore, related to fibrosis of the capsular and peri-capsular tissues in adhesive capsulitis. In addition to the direct infection of the cells outside of the respiratory tract, COVID-19 is characterized by indirect effects resulting from the host’s response to the viral infection. These indirect effects are associated with a cytokine storm and systemic inflammation that may impact nearly every organ system, including the musculoskeletal tissues.

In strong similarity to COVID-19, the inflammatory cascade implicated in abnormal tissue repair and fibrosis of the shoulder AC is supported by similar cytokines and growth factors, particularly IL-1, IL-6, and TNF-α [5]. Moreover, several authors have shown that expression levels of TNF-α, IL-1, and IL-6 are high in the joint capsule, in the subacromial bursa, and in joint fluid in patients with frozen shoulder [5,6]. These data support the hypothesis that indirect effects of viral infection may be involved in the development of adhesive capsulitis. Adhesive capsulitis is a painful shoulder condition commonly known as “frozen shoulder”. Patients suffering from this pathology present a progressive loss of range of motion (ROM) at the level of the glenohumeral joint. This condition results from progressive fibrosis and the possible contracture of the glenohumeral joint capsule, which causes pain and stiffness [7,8,9].

Adhesive capsulitis is prevalent in approximately 2% of the general population. The causes that could lead to this pathological condition can be idiopathic or traumatic (shoulder dislocation, repeated trauma, and humeral fractures) [10]. As regards etiopathogenesis, there are some predisposing diseases, such as type 1 and 2 diabetes, hyperthyroidism, hypothyroidism, Parkinson’s disease, heart disease, and autoimmune diseases [11,12].

Clinical examination and instrumental imaging are both used in diagnosing adhesive capsulitis.

Ultrasonographic signs of adhesive capsulitis consist of a thickening of the inferior recess of the glenohumeral joint capsule on a longitudinal sub-axillary scan, thickening of the coracohumeral ligament and soft tissue structures in the rotator cuff interval, subacromial-subdeltoid bursa with hypervascularization, and inflammation of the bicep tendon sheath [13,14,15,16,17].

Hydrodilatation is an effective therapeutic intervention that leads to rapid joint improvement in patients with adhesive capsulitis. This technique consists of injecting a saline solution or a saline solution combined with corticosteroids and anesthetic, which relaxes the capsule because it increases hydrostatic pressure (also called hydrodistension) and thus the volume capacity of the shoulder [18,19,20,21]. This can be carried out under fluoroscopic guidance or with ultrasound guidance, and both methods have similar outcomes [22]. However, ultrasound-guided hydrodilatation has the advantage of being fast and inexpensive and it allows an assessment of the rotator cuff muscles. Hydrodistension was first described by Andren and Lundberg, who described injection into the glenohumeral joint under X-ray guidance [23]. A Cochrane review on the effectiveness and safety of hydrodistension based on five trials, involving only one of high quality, found that the procedure may improve pain at three weeks and disability up to 12 weeks. It was concluded that there was evidence for distension with saline and steroid, providing short-term benefits in pain and range of motion in frozen shoulder [24]. With regard to a study by Ladermaan et al., the most important finding in this overview of meta-analyses on the conservative treatment of frozen shoulder was that capsular distension (hydrodilatation) with corticosteroid provided the best overall prospect for short-term pain relief and improvement in range of motion across all time frames [25].

Regarding rehabilitation, interventions performed by physiotherapists are commonly used and often recommended to treat adhesive capsulitis of the shoulder. Traditional physiotherapy consists of patient education, physical applications such as joint mobilization, and exercises [26,27]. As part of a multimodal program, the most common types of exercises were isometric or strengthening exercises for the rotator cuff, trapezius, scapular, and glenohumeral muscles, muscle energy technics (proprioceptive neuromuscular facilitation), wand/wall exercises, (Codman) pendulum exercises and stretching exercises [28,29]. The goal of exercise is to improve ROM and muscle function by restoring shoulder mobility and stability through range of motion.

In this study, we reported 16 vaccinated patients who developed adhesive capsulitis (phase 1) during the post-COVID-19 period and were treated with infiltrative hydrodistension therapy associated with early rehabilitation treatment [30,31]. The study assessed the feasibility and effectiveness of ultrasound-guided hydrodistension associated with a rehabilitation protocol for patients with adhesive capsulitis developed in post-COVID-19 syndrome.

## 2. Methods

Sixteen consecutive patients referred to the outpatient clinic for adhesive capsulitis (phase 1) in post-COVID-19 syndrome were clinically and ultrasonographically evaluated. Patients were screened based on age (between 18 and 65) and the presence of clinical and ultrasound signs of adhesive capsulitis developed within 4 months of COVID-19 infection. The exclusion criteria consisted of patients who developed adhesive capsulitis of the shoulder where they received the COVID-19 vaccination. Among the patients enrolled in the study, 10 were women and 6 were men. The mean age of the patients was 52 ± 9.53. Patients had no other concomitant pathologies such as diabetes or hyperthyroidism, and all had received double vaccination against SARS-CoV-2. Six patients had developed adhesive capsulitis on the left shoulder and ten received this on the right shoulder. Nine patients were manual laborers and seven were office workers. Adhesive capsulitis developed between 1.5 and 3 months after the COVID-19 diagnosis (mean time to onset was 2 months). In 7 of the patients, COVID-19 was asymptomatic, whereas in the remaining 9 patients, symptoms were mild. None of the patients were severely or critical ill. Patient demographics are shown in Table 1.

### 2.1. Ethical Issues

This interventional study was approved by the local ethics committee (379/2022/Sper/IOR, and approved date: 20/07/2022). The study was conducted in accordance with the ethical standards of the Declaration of Helsinki, and informed consent was obtained from all patients, including approval for photographic and/or video documentation.

### 2.2. Statistical Analysis

All continuous variables are expressed in terms of mean ± standard deviation (SD) and range. Categorical variables are summarized in terms of absolute frequency and percentage. Correlations between the means of outcome measures pre-treatment and post-treatment were analyzed with a *t*-test; *p* < 0.05 was considered significant. The statistical analysis was performed using the statistical package for social sciences (SPSS), software version 15.0 (SPSS Inc., 199 Chicago, IL, USA) by a statistical consultant from Rizzoli Orthopaedic Institute.

### 2.3. Outcome Measures

Active shoulder ROM, visual analogue scale for pain (VAS), the Shoulder Pain and Disability Index (SPADI) and the Disabilities of the Arm, Shoulder and Hand (DASH) questionnaire were used for clinical assessment. Data were recorded prior to the hydrodistension procedure and at 5 weeks and at 10 weeks post-treatment. The visual analogue scale (VAS) is a one-dimensional scale that evaluates the intensity of pain. The scale ranges from 0 to 10, with 0 being no pain and 10 being the greatest possible pain [32]. The minimal clinically important difference (MCID) is reported to be 2 for the VAS. The SPADI scale comprises a series of 13 items (5 for pain and 8 for function), each scored with a visual analogue scale ranging from 0 (no pain/no difficulty) to 10 (worse pain imaginable/so difficult that help is required). The percentage score can vary from 0 to 130 (0, painless shoulder activity and allowed; 130, painful shoulder activity and not granted) [33]. The MCID for the SPADI is reported to be 10. The DASH scale includes 21 items for a total of 105 points. This scale especially concerns the limitations in carrying out activities of daily living. The score given for each item can be 1 (no restrictions), 2 (slight limitations), 3 (moderate limitations), 4 (severe limitations), or 5 (unable). The percentage score can vary from a minimum of 0 to a maximum of 100. As the score increases, there is an increase in the patient’s inability in carrying out activities of daily living [34]. The minimal clinically important difference (MCID) is reported to be 14 for the DASH questionnaire.

### 2.4. Ultrasound Diagnosis

Ultrasound examinations of the shoulder were performed using Samsung HM70A with Plus Ultrasound System, equipped with a 5–14 MHz linear transducer with a musculoskeletal preset. All the patients were evaluated by experts of US-Diagnosis.

The following parameters were assessed:

(a) Coracohumeral ligament (CHL): for CHL assessment, patients were scanned in a sitting position, with the shoulder in a neutral position and the forearm extended. The scanning commenced in an axial oblique plane via positioning the transducer on the lateral border of the coracoid process, obtaining a longitudinal image of the CHL.
(b) Rotator interval: the rotator interval is a triangular space in the anterosuperior rotator cuff bounded above by the anterior free edge of the supraspinatus tendon and below by the superior edge of the subscapularis tendon. The rotator interval was optimally visualized in the oblique plane with the patient’s fist held by their side.

**Figure 1:**
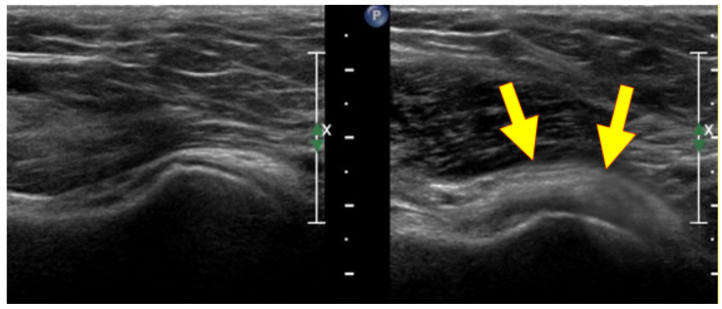
Thickening of the inferior recess of the glenohumeral joint capsule.

**Figure 2:**
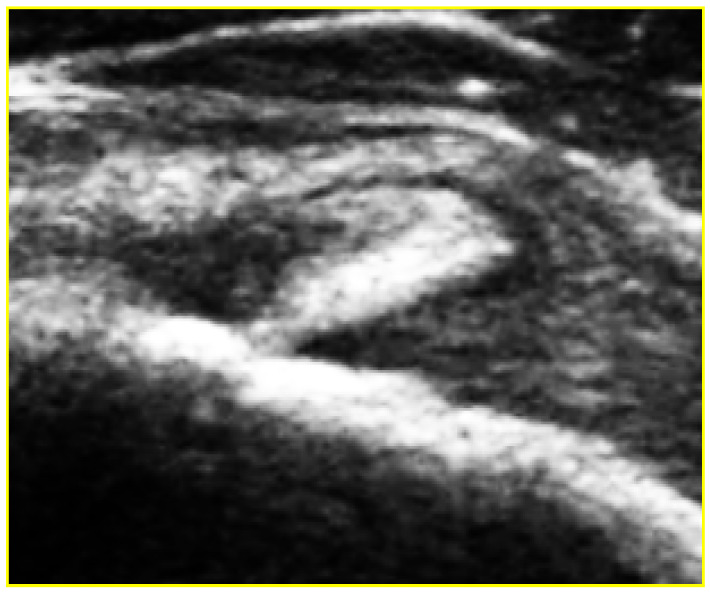
Thickening of the coracohumeral ligament with inflammation of the bicep tendon sheath.

The criteria for the ultrasound diagnosis of adhesive capsulitis were a thickening of the inferior recess of the glenohumeral joint capsule, the performance of a longitudinal subaxillary scan (Figure 1), a thickening of the coracohumeral ligament and soft tissue structures in the range of the rotator cuff, a subacromial–subdeltoid bursa with hypervascularization and inflammation of the bicep tendon sheath (Figure 2) [14,15,16,17].

### 2.5. Ultrasound-Guided Infiltrative Treatment

**Figure 3:**
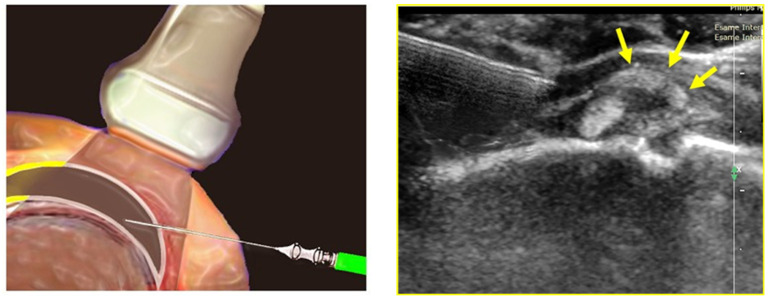
Ultrasound-guided infiltrative treatment.

Patients were treated with infiltrative hydrodistension therapy under ultrasound guidance. This technique consists of injecting a saline solution or a saline solution combined with corticosteroids and anesthetic which relaxes the capsule because it increases hydrostatic pressure (also called hydrodistension), thus increasing the volume capacity of the shoulder [18,19]. An expert musculoskeletal sonographer, using a 5–14 MHz intraoperative high-frequency linear transducer, performed ultrasound-guided anterior rotator interval hydrodilatation using 1 mL of cortisone (Depo-Medrol 40 mg), 5 mL of 2% lidocaine hydrochloride, and 4 mL of saline (Figure 3) [35,36]. All injections were performed using a 90 mm long 21 G needle, after disinfecting the skin with a solution of povidone iodine or chlorhexidine, depending on any skin allergies or intolerances.

### 2.6. Early Rehabilitation Treatment

**Figure 4:**
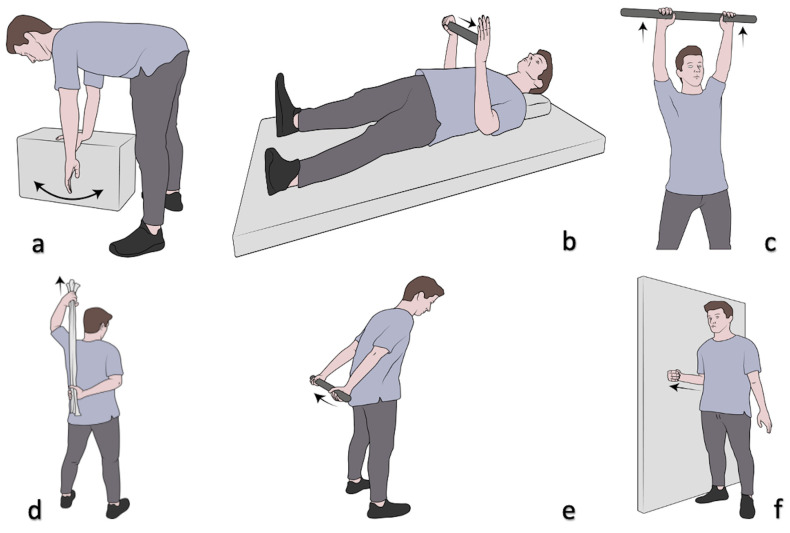
(**a**–**f**) Physiotherapy exercises with personal rehabilitation protocol (see text for details).

We adopted a rehabilitation protocol with exercises aimed at recovering range of motion, avoiding manual loads for at least the first three months. Rehabilitation started immediately after the ultrasound-guided hydrodistension infiltrative treatment to improve the joint ROM of the affected glenohumeral joint right away [26,29]. Rehabilitation was managed by two physiotherapists of physical and rehabilitation medicine at Rizzoli Orthopedic Institute who evaluated all patients before the hydrodistension procedure. The rehabilitation protocol consisted of a series of pendular exercises and passive/active mobilization exercises for the glenohumeral joint, and was carried out in 2 sessions for about 30 min per week for a total of 10 weeks (total 20 sessions), with the following exercises being performed:

- Commuting exercises: The patient bends the torso forward so that it is parallel to the floor and leans on a stool or table with the sound arm. They swing the affected limb back and forth for about 5 min. With the treated limb, the patient makes circles outwards with the palm facing out, and then circles inwards with the palm (Figure 4a).
- External rotation exercises: The patient is supine, with the arm close to the body, and the elbow flexed at 90°. The patient holds a stick with the healthy limb and places it on the palm of the affected limb, pushing on it so as to hyper-rotate the affected limb. The patient maintains this position for about 15–20 s and then returns to the starting position (Figure 4b).
- Anterior elevation exercises: Standing upright, the patient holds a cane with both hands, raises their arms above their head as far as they can, holding the position for about 10 s and then returns to the starting position (Figure 4c).
- Internal rotation exercises: The patient places the treated limb behind their back with the elbow bent. Using a towel and using the healthy limb, they bring the treated limb to the maximum internal rotation, maintaining this position for about 5 s (Figure 4d).
- Retroposition exercises: Standing upright, the patient grasps a stick behind their back with both hands and brings their shoulders to the maximum retroposition, maintaining this position for 5 s (Figure 4e).
- Abduction exercises: Standing next to a wall with the elbow flexed at 90°, the patient pushes their elbow and forearm against the wall (Figure 4f).

Patients were reviewed by two physiotherapist at 5 weeks (after 10 sessions) and at 10 weeks (after 20 sessions), and the data of the outcome measures were recorded.

## 3. Results

Sixteen patients with post-COVID-19 syndrome treated with ultrasound-guided infiltration associated with early rehabilitation treatment showed major improvement in ROM after 10 weeks, especially in shoulder elevation and abduction movements. In 16 patients with phase-1 adhesive capsulitis, before the treatment, a fairly reduced shoulder range of motion was seen with mean elevation values allowed to reach 55°, abduction allowed to reach 40°, internal rotation allowed to reach 30° with the arm abducted to 90°, and external rotation allowed to reach 40° with the arm abducted to 90°. After the infiltration treatment and the first 10 sessions of physiotherapy, there was an increase in the range of motion of the shoulder with elevation allowed to reach 100°, abduction allowed to reach 90°, internal rotation allowed to reach 50° with the arm abducted to 90°, and external rotation allowed to reach 70° with the arm abducted to 90°.

Given the clinical improvement, it was decided, in agreement with the patients, not to carry out further infiltration but to continue with another 10 physiotherapy sessions, obtaining an almost complete recovery of range of motion at the end of the cycle (elevation of 150°, abduction of 130°, internal rotation allowed to reach 70° with the arm abducted to 90°, and external rotation allowed to reach 80° with the arm abducted at 90°). The data are reported in Table 2. Regarding the SPADI scale, before treatment patients had a mean score of 55.5% ± 16.12. After the first 10 sessions of treatment, the mean was 15.4% ± 4.91. After the second set of treatment sessions, it was 8.5% ± 1.77. Additionally, the values of the DASH scale and of the VAS showed marked improvement. Before treatment, the mean DASH score was 58 ± 5.47 and the mean VAS score was 6.9 ± 1.66. After the first 10 sessions of rehabilitation, the mean DASH score was 17 ± 4.56, and the mean VAS was 1.5 ± 1.04. After the next 10 sessions, the mean DASH score was 12 ± 2.89 and the mean VAS was 1 ± 0.63. The data are reported in Table 2.

Pairwise comparisons with the *t*-test showed significant improvements between pre-treatment and after 10 sessions, between pre-treatment and after 20 sessions, and between after 10 sessions and after 20 sessions for SPADI (*p* < 0.00001), between pre-treatment and after 10 sessions, between pre-treatment and after 20 sessions (*p* < 0.00001), between after 10 sessions and after 20 sessions (*p* < 0.00002) for DASH, and between pre-treatment and after 10 sessions, between pre-treatment and after 20 sessions (*p* < 0.00001), and between after 10 sessions and after 20 sessions (*p* < 0.02) for VAS. In addition, significant improvements were seen between all time points for range of motion in abduction and flexion (*p* < 0.00001), between pre-treatment and after 10 sessions, between pre-treatment and after 20 sessions (*p* < 0.00001) and between after 10 sessions and after 20 sessions (*p* < 0.005) in external rotation and between pre-treatment and after 10 sessions, (*p* < 0.00002), pre-treatment and after 20 sessions, and between after 10 sessions and after 20 sessions (*p* < 0.00001) in internal rotation. The data are reported in Table 3.

## 4. Discussion

Adhesive capsulitis in subjects who have contracted COVID-19 infection may be due to an exaggerated inflammatory response linked to an infection that also involves the musculoskeletal tissues. There was only one study in the literature that tried to describe the correlation between COVID-19 infection and the subsequent development of capsulitis [1]. In the study by Ascani et al., it was shown that adhesive capsulitis in post-COVID-19 syndrome may be due to direct effects related to the ability of the virus to attack synovial cells and indirect effects related to the activation of the inflammatory cascade with the overproduction of inflammatory cytokines [1,5,6]. In the literature, there was a study of Biglia et al. that presented a case of a patient who developed adhesive capsulitis after the second dose of the SARS-CoV-2 vaccine. The patient underwent two US-guided capsule hydrodistension porceudres associated with early rehabilitation and after 4 weeks the pain resolved and ROM was almost completely recovered [37]. In this study, we treated patients affected by adhesive capsulitis (phase 1) in post-COVID-19 syndrome, proposing ultrasound-guided anterior rotator interval hydrodilatation combined with a rehabilitation treatment (protocol) with specific exercises. Hydrodilatation is an effective therapeutic intervention that leads to rapid joint improvement in patients with adhesive capsulitis, and it can be performed with ultrasound guidance [20,25]. A recent analysis of the health economy showed that ultrasound-guided capsular hydrodistension costs about ten times less than arthroscopic capsular release does. Hydrodistension can therefore provide treatment for patients with frozen shoulder that is relatively cheap, fast, easily accessible and effective [31]. In a randomized controlled study by Elnady et al., it emerged that ultrasound-guided anterior rotator interval hydrodilatation combined with a local corticosteroid for adhesive capsulitis, followed by guided exercise, is clinically and functionally more effective than the conventional posterior approach is [36]. Early rehabilitation, aimed at improving painful symptoms and joint ROM, must be immediately associated with the infiltrative hydrodistension treatment [30,31]. According to the 2017 study by Chan et al., rehabilitation consisted of the functional re-education of the glenohumeral joint with pendular exercises and passive/active mobilization exercises [26,28]. In the 2019 study by Redler et al., there was a comparison of surgical and conservative treatment in adhesive capsulitis, most patients had a complete resolution of symptoms with non-surgical management, and there appeared to be a role of early corticosteroid injection in shortening the overall duration of symptoms [38]. In particular, hydrodistension combined with early rehabilitation provided earlier pain relief and restoration of shoulder ROM and function compared to single intra-articular corticosteroid injection in patients with adhesive capsulitis. A review by the Cochrane Library showed that, in adhesive capsulitis, infiltration therapy with glucocorticoids and saline water solution associated with early rehabilitation treatment provided more successful results in terms of the passive/active ROM of the joint and the reduction in pain compared to those of infiltration treatment alone or rehabilitation [39]. In a systematic review and meta-analysis by Challoumas et al., the findings of the study suggested that the early use of an intra-articular corticosteroid in patients with frozen shoulder of less than a 1-year duration was associated with better outcomes. This treatment should be accompanied by an exercise program to maximize the chance of recovery [40]. In a systematic review of Nakandala et al., all studies evaluated the efficacy of the combination of treatment approaches despite a single treatment in terms of pain relief, the improvement of ROM and the functional status of patients with adhesive capsulitis. We concluded that all diagnoses of frozen shoulder were correct, and there were no adverse incidents or complications in the evaluation. Although this was not a randomized controlled trial, and only a small number of participants were studied, the results suggest that ultrasound-guided hydrodistension and physiotherapy-guided exercise for patients with frozen shoulder (phase 1) developed in post-COVID-19 syndrome was effective at improving pain, disability and movement. This improvement was maintained for 10 weeks for all outcome measures. The results of the present study clearly demonstrate a clinically significant change in the SPADI at both of the two time points from the baseline. Clinically, an effective treatment should result in a significant change after the first 10 sessions. The MCID for the SPADI is reported to be a 10-point change. We clearly surpassed the recommended level of change. Similarly, the MCID for the DASH questionnaire is reported to be a 14-point change. DASH results demonstrated a clinically significant sustained change at both of the two time points (Table 3). Clinically recorded outcome measures of external and internal rotation, abduction and flexion movements continued to show statistically significant improvements (*p* < 0.00001) and clinically significant changes at all time points from the baseline and between all time points, indicating a continued functional recovery of movement. The mean pain score went down significantly, from 6.9 (pre) to 1.5 (after 10 sessions) (*p* < 0.00001), showing a 78% reduction in pain after 10 sessions. This trend continued after 20 sessions to 1 (*p* < 0.00001), for an 86% reduction. These findings were confirmed in this study, in which we obtained a satisfying outcome from the treatment.

## 5. Limitations of the Study

The main limitations of the study were the limited size of the study group, although it should be borne in mind that we enrolled highly selected patients who had recently suffered from COVID-19 and who had adhesive capsulitis of the shoulder with no other apparent causes, with the absence of a control group consisting of patients with adhesive capsulitis developed in post-COVID-19 syndrome not subjected to treatment.

## 6. Conclusions

The results of this study demonstrated that ultrasound-guided hydrodistension with guided exercise provided by physiotherapists in primary care was clinically effective for patients with adhesive capsulitis (phase 1) developed in post-COVID-19 syndrome in terms of joint movement recovery and pain reduction. Although these findings did not provide new evidence on treatment efficacy, they were consistent with the previous findings of hydrodistension treatment associated with rehabilitation treatment. Future studies will be needed to compare these results given the small number of patients collected due to the recently discovered association between adhesive capsulitis and post-COVID-19 syndrome and the absence of a control group consisting of patients with adhesive capsulitis developed in post-COVID-19 syndrome not subjected to treatment.

## Figures and Tables

**Table 1:** General data.

Patient No.	Sex	Age	Side	* AC Onset	COVID-19 Severity	Job
1	F	30	L	1.5	Mild	OW
2	M	50	R	3	Asymptomatic	ML
3	F	60	L	2	Mild	ML
4	M	64	R	1.5	Asymptomatic	OW
5	F	46	R	2	Asymptomatic	ML
6	F	54	L	1.5	Mild	OW
7	M	48	R	3	Asymptomatic	OW
8	F	36	L	2	Mild	ML
9	M	52	R	1.5	Mild	ML
10	F	54	R	2	Asymptomatic	OW
11	M	62	L	3	Mild	ML
12	F	57	R	1.5	Mild	OW
13	F	43	R	2	Asymptomatic	ML
14	F	59	R	1.5	Mild	ML
15	M	56	L	2	Asymptomatic	ML
16	F	61	R	2	Mild	OW

F = female; M = male; L = left; R = right; AC = adhesive capsulitis, E = office worker, ML = manual laborer; * AC onset after COVID-19 diagnosis in terms of months.

**Table 2:** Mean results of outcome measures and (SD) pre- and post-treatment.

	Pre-Treatment Mean (SD)	After 10 Sessions Mean (SD)	After 20 Sessions Mean (SD)
Flexion (°)	55 (10)	100 (7.07)	150 (12.24)
Abduction (°)	40 (7.74)	90 (14.14)	130 (13.78)
External rotation (°)	40 (7.07)	70 (11.83)	80 (12.64)
Internal rotation (°)	30 (8.36)	50 (7.07)	70 (13.03)
SPADI	55 (16.12)	15.4 (4.91)	8.5 (1.77)
DASH	58 (5.47)	17 (4.56)	12 (2.89)
VAS	6.9 (1.66)	1.5 (1.04)	1 (0.63)

**Table 3:** Mean differences and pairwise comparisons with *t*-test.

	ROM Flexion	ROM Abduction	ROM ext. Rotation	ROM int. Rotation	SPADI	DASH	VAS
Pre to 10 sessions	13.81 *p* < 0.00001	10.35 *p* < 0.00001	6.93 *p* < 0.00001	5.82 *p* < 0.00002	−24.26 *p* < 0.00001	−25.89 *p* < 0.00001	−19.39 *p* < 0.00001
Pre to 20 sessions	32.91 *p* < 0.00001	27.89 *p* < 0.00001	10.12 *p* < 0.00001	14.41 *p* < 0.00001	−35.97 *p* < 0.00001	−29.85 *p* < 0.00001	−18.42 *p* < 0.00001
After 10 to 20 sessions	11.24 *p* < 0.00001	9.68 *p* < 0.00001	2.9 *p* < 0.005	19.6 *p* < 0.00001	−11.5 *p* < 0.00001	−5.87 *p* < 0.00002	−2.07 *p* < 0.02

## Data Availability

Not applicable.
